# The COVID-19 Ontology

**DOI:** 10.1093/bioinformatics/btaa1057

**Published:** 2020-12-21

**Authors:** Astghik Sargsyan, Alpha Tom Kodamullil, Shounak Baksi, Johannes Darms, Sumit Madan, Stephan Gebel, Oliver Keminer, Geena Mariya Jose, Helena Balabin, Lauren Nicole DeLong, Manfred Kohler, Marc Jacobs, Martin Hofmann-Apitius

**Affiliations:** Department of Bioinformatics, Fraunhofer Institute for Algorithms and Scientific Computing (SCAI), 53754 Sankt Augustin, Germany; Bonn-Aachen International Center for Information Technology (B-IT), University of Bonn, 53113 Bonn, Germany; Department of Bioinformatics, Fraunhofer Institute for Algorithms and Scientific Computing (SCAI), 53754 Sankt Augustin, Germany; Causality Biomodels, Kinfra Hi-Tech Park, Cochin, Kerala 683503, India; Department of Bioinformatics, Fraunhofer Institute for Algorithms and Scientific Computing (SCAI), 53754 Sankt Augustin, Germany; Department of Bioinformatics, Fraunhofer Institute for Algorithms and Scientific Computing (SCAI), 53754 Sankt Augustin, Germany; Department of Bioinformatics, Fraunhofer Institute for Algorithms and Scientific Computing (SCAI), 53754 Sankt Augustin, Germany; Fraunhofer Institute for Molecular Biology and Applied Ecology-ScreeningPort, Hamburg, Germany; Causality Biomodels, Kinfra Hi-Tech Park, Cochin, Kerala 683503, India; Department of Bioinformatics, Fraunhofer Institute for Algorithms and Scientific Computing (SCAI), 53754 Sankt Augustin, Germany; Department of Bioinformatics, Fraunhofer Institute for Algorithms and Scientific Computing (SCAI), 53754 Sankt Augustin, Germany; Fraunhofer Institute for Molecular Biology and Applied Ecology-ScreeningPort, Hamburg, Germany; Department of Bioinformatics, Fraunhofer Institute for Algorithms and Scientific Computing (SCAI), 53754 Sankt Augustin, Germany; Department of Bioinformatics, Fraunhofer Institute for Algorithms and Scientific Computing (SCAI), 53754 Sankt Augustin, Germany; Bonn-Aachen International Center for Information Technology (B-IT), University of Bonn, 53113 Bonn, Germany

## Abstract

**Motivation:**

The COVID-19 pandemic has prompted an impressive, worldwide response by the academic community. In order to support text mining approaches as well as data description, linking and harmonization in the context of COVID-19, we have developed an ontology representing major novel coronavirus (SARS-CoV-2) entities. The ontology has a strong scope on chemical entities suited for drug repurposing, as this is a major target of ongoing COVID-19 therapeutic development.

**Results:**

The ontology comprises 2270 classes of concepts and 38 987 axioms (2622 logical axioms and 2434 declaration axioms). It depicts the roles of molecular and cellular entities in virus-host interactions and in the virus life cycle, as well as a wide spectrum of medical and epidemiological concepts linked to COVID-19. The performance of the ontology has been tested on Medline and the COVID-19 corpus provided by the Allen Institute.

**Availabilityand implementation:**

COVID-19 Ontology is released under a Creative Commons 4.0 License and shared via https://github.com/covid-19-ontology/covid-19. The ontology is also deposited in BioPortal at https://bioportal.bioontology.org/ontologies/COVID-19.

**Supplementary information:**

Supplementary data are available at *Bioinformatics* online.

## 1 Introduction

Since the end of 2019, the COVID-19 pandemic is in the spotlight of the global scientific community. The spectrum of scientific approaches to combat SARS-CoV-2 includes diverse disciplines such as epidemiology modeling (Kucharski *et al.*, 2020, Zhao and Chen, 2020), molecular docking and molecular dynamics on supercomputers (Smith and Smith, 2020) and high throughput screening using drug-repurposing libraries (Jin *et al.*, 2020, Ton *et al.*, 2020).

One major bottleneck facing current strategies to find candidate compounds for drug repurposing is the limited availability of chemical information in the COVID-19 context. Any serious attempt at identifying new or repurposed drugs against SARS-CoV-2 needs to consider what has been published on the respective drug target and what classes of chemical compounds display activity against viral proteins. Despite that, various publications claim to have identified new candidates for drug repurposing (Fan *et al.*, 2020). The literature on COVID-19 targets and putative target-binding ligands is growing with impressive dynamics.

To facilitate dedicated literature searches on COVID-19 pathophysiology, epidemiology, targets and medical implications, we have generated a prototypical version of a COVID-19 ontology. This ontology is meant to capture and represent the majority of essential entities and concepts relevant for COVID-19 research context. The ontology serves two major purposes:

- As a template to define context in COVID-19 specific text mining approaches.
- As a structured system of concepts and categories that helps to bring order into the COVID-19 knowledge space.

We demonstrate the usability of the COVID-19 Ontology in both cases.

## 2 Material and methods

Starting from a mind map generated by a concerted action of biologists, pharmacologists and computational biologists working on COVID-19, we identified major aspects of virology, epidemiology, chemical biology and clinical aspects relevant for translational COVID-19 research. Extensive literature searches were done to enrich entities and concepts relevant for the disease. An expert-curated mind map of COVID-19 knowledge was used to establish the core structure of the ontology. Relevant entities and concepts were collected from 32 Research articles, 10 Reviews, as well as from various relevant websites such as WHO (https://www.who.int/), Radiology Assistant COVID-19 (https://radiologyassistant.nl/chest/lk-jg-1), Centre for Evidence-Based Medicine (https://www.cebm.net/covid-19/registered-trials-and-analysis), Texas Medical Center (https://www.tmc.edu/news/2020/03/covid-19-crisis-catalog-a-Glossary-of-terms), Yale Medicine (https://www.yalemedicine.org/stories/covid-19-glossary/), Targeting COVID-19: GHDDI Info Sharing Portal (https://ghddi-ailab.github.io/Targeting2019-nCoV), Summit Medical Group (https://www.summitmedicalgroup.com/news/living-well/must-know-Covid-19-vocabulary) and Georgetown University (https://www.georgetown.edu/Coronavirus/glossary-of-terms). Additionally, we have also included some of the openly available vocabularies, e.g. (https://github.com/SciBiteLabs/CORD19/) as well as a manually curated version of n-grams (bi- and trigrams), representing a list of terms co-occurring most frequently, generated using 2170 abstracts from the LitCovid database as of April 1, 2020 (see Supplementary Document).

The COVID-19 Ontology was assembled using the Protégé ontology editor. This ontology is constructed based on guidelines and principles defined by Open Biological and Biomedical Ontology (OBO, http://www.obofoundry.org/) foundry as well as aligned with the Basic Formal Ontology (BFO) hierarchy. We applied Ontofox (http://ontofox.hegroup.org) to reuse already existing classes from other relevant ontologies. Terms not predefined in other ontologies are added with proper definitions as well as clear provenance. In order to increase recall in text mining applications, we have added synonyms for each concept. The COVID-19 Ontology is released under a Creative Commons 4.0 License and shared via https://github.com/covid-19-ontology/covid-19. The ontology is also deposited in BioPortal at https://bioportal.bioontology.org/ontologies/COVID-19 and in the COVID-OLS at https://ols-covid.scaiview.com/ols/ontologies/covid19. The version made available here will be constantly updated. We would like to encourage the community to help us improve the ontology by providing feedback and application examples.

## 3 Results

The COVID-19 Ontology comprises 2270 terms, including 2121 terms imported from existing ontologies together with 149 newly defined terms. The ontology focuses on a wide spectrum of domain-specific topics ranging from epidemiology (risk factors, transmission, etc.), via clinical aspects (such as signs and symptoms, diagnostics, medical intervention) and aspects of prevention and control, to clinical trials, genetic and molecular processes (of both human and virus) and signaling pathways.

We are aware of the parallel development of ontologies (see Supplementary Document for comparison with other related ontologies) around COVID-19, however, many of them were not suitable for the use cases we define below.

### 3.1 Application in text mining

From the COVID-19 Ontology, we have derived a terminology tailored for use in text mining. For named entity recognition, concepts and entities were used together with their synonyms. The terminology was then integrated into the literature mining engine SCAIView (https://covid.scaiview.com/) and tested in retrieval and entity recognition experiments. Adaptation of the ontology for text mining purposes allows for information retrieval and information extraction of COVID-related research topics such as ‘risk factor’, ‘clinical aspect’, ‘prevention and control’, ‘model’, ‘transmission process’, ‘signs and symptoms’, ’virology’. The aforementioned topics are defined via logical Axioms. This allows grouping of related concepts and classes. This is used in the search engine SCAIView to aggregate related research documents into topics (see Supplementary Document). The text mining service will be made publicly available in a dedicated, public SCAIView COVID environment.

### 3.2 Organizing the COVID-19 knowledge space

The ontology is used to annotate data and models in the COVID-19 Knowledge Space (https://www.covid19-knowledgespace.de). We are using the COVID-19 ontology for efficient data interchange, data sharing and semantic interoperability among different sources in the COVID-19 Knowledge Space. One of the core components of the COVID-19 Knowledge Space is the Covid Knowledge SuperGraph (https://www.covid19-knowledgespace.de/), which is built by integrating knowledge from literature, publicly available knowledge graphs and disease maps including proprietary pathophysiology graphs (https://precisionlife.com/news/Covid19-sepsis/). COVID-19 Ontology is used to attain the semantic interoperability and mapping between various entities and relationships in the COVID Knowledge SuperGraph.

## 4 Discussion

The novel coronavirus has been the prime focus of thousands of scientific research topics for almost several months. Numerous scientific publications are published, and dealing with that much information brings growing limitations for the researchers and clinicians, and it is crucial to implement tools to overcome the mentioned issue. The developed COVID-19 Ontology captures and represents the essential entities and concepts relevant for COVID-19 research, and is meant to assist text mining approaches and semantic interoperability in the COVID-19 domain. COVID-19 Ontology mentioned here forms the basis for establishing the reference namespace for the COVID Knowledge Graph that encapsulates the molecular mechanisms around COVID-19 infection (Domingo-Fernández *et al.*, 2020). With the text mining use case described in our paper, researchers and clinicians have the opportunity to find papers more specific to their interests. It is also noteworthy to mention that our ontology is well suited to categorize and annotate the rapidly growing number of COVID-19 portals.

## Supplementary Material

btaa1057_Supplementary_Data

## References

[btaa1057-B1] Domingo-Fernández. et al (2020) COVID-19 Knowledge Graph: a computable, multi-modal, cause-and-effect knowledge model of COVID-19 pathophysiology, *Bioinformatics*, btaa834, https://doi.org/10.1093/bioinformatics/btaa834 10.1093/bioinformatics/btaa834 PMC7558629 32976572

[btaa1057-B2] Fan H.H. et al (2020) Repurposing of clinically approved drugs for treatment of coronavirus disease 2019 in a 2019-novel coronavirus (2019-nCoV) related coronavirus model. Chin. Med. J. (Engl.). 10.1097/CM9.0000000000000797 PMC7147283 32149769

[btaa1057-B3] Jin Z. et al (2020) Structure-based drug design, virtual screening and high-throughput screening rapidly identify antiviral leads targeting COVID-19 bioRxiv. doi:10.1101/2020.02.26.964882.

[btaa1057-B4] Kucharski A.J. et al (2020) Early dynamics of transmission and control of COVID-19: a mathematical modelling studyLancet. Infect Dis. doi: 10.1016/S1473-3099(20)30144-4 10.1016/S1473-3099(20)30144-4 PMC7158569 32171059

[btaa1057-B5] Smith M. , Smith J.C. (2020) Repurposing Therapeutics for COVID-19: Supercomputer-Based Docking to the SARS-CoV-2 Viral Spike Protein and Viral Spike Protein-Human ACE2 Interface. ChemRxiv. doi:10.26434/chemrxiv.11871402.v4

[btaa1057-B6] Ton A.T. et al (2020) 2020 rapid identification of potential inhibitors of SARS-CoV-2 main protease by deep docking of 1.3 billion compounds. Mol. Inform., 39, 2000028. 32162456 10.1002/minf.202000028 PMC7228259

[btaa1057-B7] Zhao S. , Chen H. (2020) Modeling the epidemic dynamics and control of COVID-19 outbreak in China. Quant. Biol., 8, 11–19. 32219006 10.1007/s40484-020-0199-0 PMC7095099

