# Supplementary material for: The COVID-19 Ontology
Source: Bioinformatics. 2020 Dec 21;36(24):5703–5. doi: 10.1093/bioinformatics/btaa1057 (PMC7799333; doi:10.1093/bioinformatics/btaa1057)
Supplement: btaa1057_Supplementary_Data [file btaa1057_supplementary_data.pdf]

Supplementary Document

Building of the ontology

Collection of Concepts:

In order to build the content of the COVID-19 Ontology, we have done manual searches of various COVID-19 or SARS-CoV-2 related websites, resources, and publications. Below is the list of various resources:

- Initially, an expert-curated mind map of COVID-19 knowledge was used to establish the core structure of the ontology.
- Relevant entities and concepts were collected from 32 research articles and 10 reviews. Below is the list of PMIDs and DOIs of articles from which we have collected the concepts.

Number	Article/Review	PMID/DOI	Title
1.	Article	PMID: 32251767	Remdesivir, lopinavir, emetine, and homoharringtonine inhibit SARS-CoV-2 replication in vitro
2.	Article	PMID: 32081636	Severe acute respiratory syndrome coronavirus 2 (SARS-CoV-2) and coronavirus disease-2019 (COVID-19): The epidemic and the challenges
3.	Article	DOI: https://doi.org/10.26434/chemrxiv.11860011.v2	Potential inhibitors against papain-like protease of novel coronavirus (SARS-CoV-2) from FDA approved drugs
4.	Article	PMID: 32363136	Crystal structure of SARS-CoV-2 nucleocapsid protein RNA binding domain reveals potential unique drug targeting sites
5.	Article	PMID: 32511329	A SARS-CoV-2-Human Protein-Protein Interaction Map Reveals Drug Targets and Potential Drug-Repurposing
6.	Article	PMID: 32838362	Virus-Host Interactome and Proteomic Survey Reveal Potential Virulence Factors Influencing SARS-CoV-2 Pathogenesis

7.	Article	PMID: 32604404	Suppression of a SARS-CoV-2 outbreak in the Italian municipality of Vo'
8.	Article	PMID: 32253318	Effectiveness of convalescent plasma therapy in severe COVID-19 patients
9.	Article	PMID: 31233808	Broad spectrum antiviral remdesivir inhibits human endemic and zoonotic deltacoronaviruses with a highly divergent RNA dependent RNA polymerase
10.	Article	PMID: 32194980	Network-based drug repurposing for novel coronavirus 2019-nCoV/SARS-CoV-2
11.	Article	PMID: 32147628	Discovering drugs to treat coronavirus disease 2019 (COVID-19)
12.	Article	PMID: 32289214	Spread of SARS-CoV-2 in the Icelandic Population
13.	Article	DOI: 10.20944/preprints202003.0286.v1	Network bioinformatics analysis provides insight into drug repurposing for COVID-2019
14.	Article	PMID: 32275855	Structural and Functional Basis of SARS-CoV-2 Entry by Using Human ACE2
15.	Article	PMID: 32171076	Clinical course and risk factors for mortality of adult inpatients with COVID-19 in Wuhan, China: a retrospective cohort study
16.	Article	PMID: 32192578	COVID-19: consider cytokine storm syndromes and immunosuppression
17.	Article	DOI: 10.20944/preprints202003.0446.v1	Systematic Cell Line-Based Identification of Drugs Modifying ACE2 Expression
18.	Article	DOI: 10.20944/preprints202004.0062.v1	Screening of FDA approved drugs against COVID-19 Main Protease: Coronavirus disease
19.	Article	PMID: 32179150	Teicoplanin: an alternative drug for the treatment of COVID-19?

20.	Article	DOI: 10.1101/2020.02.05.935387	Teicoplanin potently blocks the cell entry of 2019-nCoV
21.	Article	PMID: 31987001	Genomic characterization of the 2019 novel human-pathogenic coronavirus isolated from a patient with atypical pneumonia after visiting Wuhan
22.	Article	PMID: 32245346	The Coronavirus and the Risks to the Elderly in Long-Term Care
23.	Article	DOI: https://doi.org/10.1101/2020.02.27.20027557	Therapeutic effects of dipyridamole on COVID-19 patients with coagulation dysfunction
24.	Article	PMID: 32284616	Estimating clinical severity of COVID-19 from the transmission dynamics in Wuhan, China
25.	Article	PMID: 32052514	The COVID-19 epidemic
26.	Article	PMID: 32228226	Transcriptomic characteristics of bronchoalveolar lavage fluid and peripheral blood mononuclear cells in COVID-19 patients
27.	Article	PMID: 32125455	Angiotensin-converting enzyme 2 (ACE2) as a SARS-CoV-2 receptor: molecular mechanisms and potential therapeutic target
28.	Article	PMID: 32204755	SARS-CoV-2 infection with gastrointestinal symptoms as the first manifestation in a neonate
29.	Article	PMID: 32191259	Characteristics and Outcomes of 21 Critically Ill Patients With COVID-19 in Washington State
30.	Article	PMID: 32142651	SARS-CoV-2 Cell Entry Depends on ACE2 and TMPRSS2 and Is Blocked by a Clinically Proven Protease Inhibitor
31.	Article	PMID: 32112884	Clinical characteristics and imaging manifestations of the 2019 novel coronavirus disease (COVID-19):A multi-center study in Wenzhou city, Zhejiang, China

32.	Article	PMID: 32183941	A Sequence Homology and Bioinformatic Approach Can Predict Candidate Targets for Immune Responses to SARS-CoV-2
33.	Review	PMID: 32052466	Potential interventions for novel coronavirus in China: A systematic review
34.	Review	PMID: 32257431	COVID-19 infection: Origin, transmission, and characteristics of human coronaviruses
35.	Review	PMID: 32113704	The epidemiology and pathogenesis of coronavirus disease (COVID-19) outbreak
36.	Review	PMID: 32166607	A Review of Coronavirus Disease-2019 (COVID-19)
37.	Review	PMID: 32134278	Perspectives on monoclonal antibody therapy as potential therapeutic intervention for Coronavirus disease-19 (COVID-19)
38.	Review	PMID: 25304691	Coronavirus-induced ER stress response and its involvement in regulation of coronavirus-host interactions
39.	Review	PMID: 32020915	The Novel Coronavirus: A Bird's Eye View
40.	Review	PMID: 32141569	COVID-19 (Novel Coronavirus 2019) - recent trends
41.	Review	PMID: 24987391	Coronavirus infection, ER stress, apoptosis and innate immunity
42.	Review	PMID: 32216698	Elevated Plasmin(ogen) as a Common Risk Factor for COVID-19 Susceptibility

Supplementary Table 1: Research articles and reviews considered to extract the main concepts

- Additionally, we have also done thorough searches of various relevant websites to extract the concepts:

**Websites**	**URL**
WHO	https://www.who.int/
Radiology Assistant COVID-19	https://radiologyassistant.nl/chest/lk-jg-1
Centre for Evidence-Based Medicine	https://www.cebm.net/covid-19/registered-trials-and-analysis
Texas Medical Center	https://www.tmc.edu/news/2020/03/covid-19-crisis-catalog-a-Glossary-of-terms
Yale Medicine	https://www.yalemedicine.org/stories/ covid-19-glossary/
Targeting COVID-19: GHDDI Info Sharing Portal	https://ghddi-ailab.github.io/Targeting2019-nCoV
Summit Medical Group	https://www.summitmedicalgroup.com/news/living-well/must-know-covid-19-vocabulary
Georgetown University	https://www.georgetown.edu/Corona virus/glossary-of-terms
SciBiteLabs (Github)	https://github.com/SciBiteLabs/ CORD19/

Supplementary Table 2: Websites considered to collect concepts

- We have also manually curated n-grams (bi- and trigrams), representing a list of terms co-occurring most frequently within 2170 abstracts from the LitCovid database as of April 1st, 2020.

Construction of the ontology

The COVID-19 Ontology was assembled using the Protégé ontology editor. This ontology is constructed based on guidelines and principles defined by Open Biological and Biomedical Ontology (OBO, http://www.obofoundry.org/) Foundry as well as aligned with the Basic Formal Ontology (BFO) hierarchy. Wherever possible, the hierarchy was based on the parent ontology from which the concept was imported. We applied Ontofox (http://ontofox.hegroup.org) to reuse previously existing classes from other relevant ontologies. For each concept, most of the OBO ontology sources were identified, and the concepts were imported with all available annotations via Ontofox. Undefined terms in other ontologies are added with proper definitions as well as with clear provenance. In order to increase recall in Text Mining applications, we have added synonyms for each concept.

Metadata information using annotation properties

We have added quite a lot of metadata information using various annotation properties. Annotation properties are added to each entity in the ontology. These properties explain the name of the entity, standard ontology from which the entity is imported, definition of the term, source from which the term is adopted, and references that connect the term to COVID-19. The details of the annotation properties are listed below:

- **label:** label annotation denotes the display name of the concept
- **oboInowl:hasDefinition:** Definitions are added under this annotation property
- **rdfs:isDefinedBy:** Used to point to the source of the definition when the definition is added manually. For modified definitions, source of the definition is given under oboInOwl:hasDbXRef or rdfs:seeAlso
- **oboInOwl:hasDbXRef:** To add additional link from Pubmed/NCBI
- **rdfs:seeAlso:** Any additional relevant links like web articles are given under rdfs:seeAlso
- **oboInOwl:hasExactSynonym:** Synonyms are added under this annotation property. Source of the synonyms are the terms from articles or research papers
- **oboInOwl:hasRelatedSynonym:** The terms that are related synonyms of the entity are added under this annotation property

Moreover, in regards to the aforementioned annotation properties, we have added some custom annotation properties to define additional information as well as to aid in applying the ontology for the use cases defined in the manuscript. Some custom annotations are used to mention the source from which the terms are taken, specifying if the definition is modified from the source article of the definition and giving an explanation of the BFO hierarchy of the term. These custom annotations are listed below:

- **fromSciBite** - concepts are taken from SciBite resource (https://github.com/SciBiteLabs/CORD19/)
- **fromBEL** - concepts are taken from COVID-19 BEL (Biological Expression Language) scripts available at https://github.com/covid19kg
- **fromPubMed** - concepts are taken from PubMed article
- **fromArticle** - concepts are taken from other resources than PubMed
- **fromNCBIBook** - concepts are taken from NCBI Books
- **CommentonDefinition** - to mention that given definition taken from the source is modified
- **CommentonHierarchy** - to explain the integration of the concept into the given hierarchy
- **DrugsinVirtualScreening** - to mention the drugs suggested in virtual screening

Hierarchical structure of the COVID -19 Ontology

COVID-19 Ontology is a hybrid of multiple OBO Foundry ontology terms and new COVID-19 specific terms. In general, Basic Formal Ontology (BFO) (http://basic-formal-ontology.org/) acts as the top level ontology. Terms from existing Open Biological and Biomedical Ontology (OBO) ontologies such as OBI, OGMS, HP, and GO were integrated by the Ontofox tool[1] in

accordance to the OBO Foundry[2] principles and the minimum information to reference an external ontology term (MIREOT) principles[3], whenever possible (Supplementary Figure S1).

COVID-19 Ontology specific terms are more terminal, comprising leaves or parents with a short distance to the leaves. OBO ontologies terms are slightly more central or internal, bridging BFO and COVID-19 Ontology, but they could also be very bottom (e.g., GO related concepts).

Text Mining related BINS are located in a sidechain outside the BFO hierarchy. Those concepts enable a collection of search terms for Text Mining applications from different branches. The concepts are related to the Text Mining BINs via specific axioms (relations). Please note that the term 'risk factor' has only 6 child terms, whereas the class 'COVID risk factor' integrates 142 concepts from different hierarchical areas of the ontology.

Most concepts are in relation to 'material' entities including concepts related to genes (196), chemicals (including drugs) (260), and taxonomy (318), followed by phenotypically abnormality (153), disposition (including disease) (127), and symptoms (119). These are all located under the BFO concept 'specifically dependent continuant'. Many processual concepts are located under 'planned process' (OBI), including relevant viral (110) and bodily processes (105) and concepts that are related to clinical aspects such as treatment (6), intervention (30)

1 Xiang Z, Courtot M, Brinkman RR, Ruttenberg A, He Y. OntoFox: web-based support for ontology reuse. *BMC Research Notes*. 2010, 3:175.

2 Smith B, Ashburner M, Rosse C, Bard J, Bug W, Ceusters W, et al. The OBO Foundry: coordinated evolution of ontologies to support biomedical data integration. Nat Biotechnol. 2007;25(11):1251–5.

3 Courtot M, Gibson F, Lister AL, Malone J, Schober D, Brinkman RR, et al. MIREOT: The minimum information to reference an external ontology term. *Applied Ontology*. 2011;6(1):23–33.

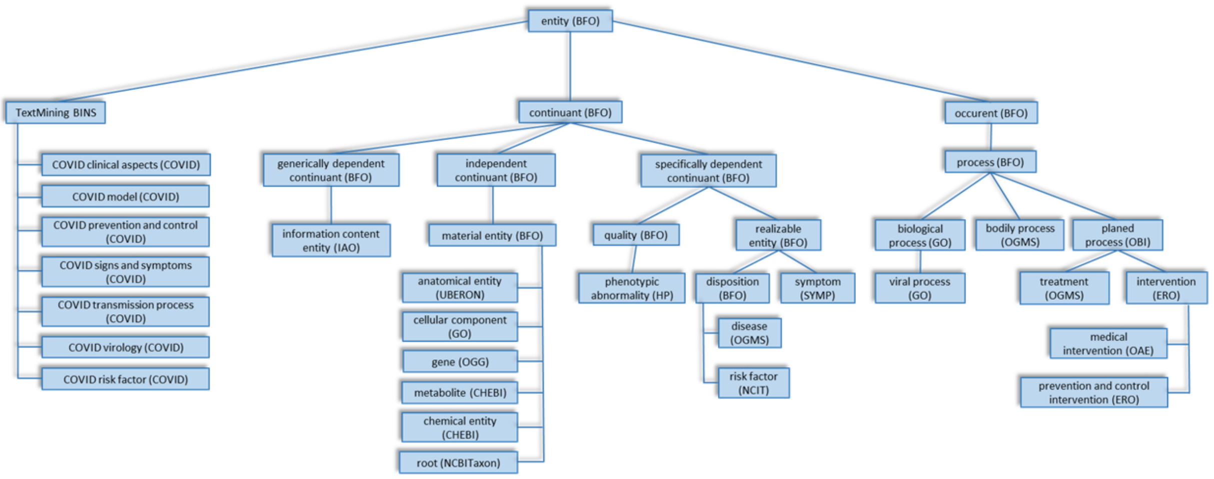

Supplementary Figure 1: Top hierarchical structure of COVID-19 Ontology.

Comparison to other ontologies

In early 2020, during the beginning of the COVID-19 pandemic, many research groups from all over the world started with various efforts against COVID-19, but only one other group began developing COVID-19 related ontologies as early as we did. This section describes the comparison of COVID-19 Ontology we have developed with the other related COVID-19 or SARS-CoV-2 ontologies.

Name	Scope	Total no. of Concepts	No. of Common Concepts	No. of Uncommon Concepts	Publication Reference
**COVID-19**<br><br>*(Ontology referred in the manuscript, version: Version Release: 1.0.0)*	Ontology covers the role of molecular and cellular entities in virus-host-interactions, in the virus life cycle, as well as a wide spectrum of medical and epidemiological concepts linked to COVID-19.	2268	-	-	-
**COVID19** - COVID-19 Surveillance Ontology (version: V0.2)	Developed to support COVID-19 (Wuhan novel Coronavirus infection) surveillance in primary care.	52	1	51	de Lusignan S, Liyanage H, McGagh D, Jani BD, Bauwens J, Byford R, Evans D, Fahey T, Greenhalgh T, Jones N, Mair FS, Okusi C, Parimalanathan V, Pell JP, Sherlock J, Tamburis O, Tripathy M, Ferreira F, Williams J, Hobbs FR<br>In-pandemic development of an application ontology for COVID-19 surveillance in a primary care sentinel network<br>JMIR Preprints. 01/07/2020:21434<br>https://preprints.jmir.org/preprint/21434
**COVIDCRFRAPID** - WHO COVID-19 Rapid Version CRF semantic data model (version: 1.1.4)	A semantic data model for the WHO's COVID-19 case record form RAPID version, that aims at providing semantic references to the questions and answers of the form.	398	46	352	D. Kringos, F. Carinci, E. Barbazza, V. Bos, K. Gilmore, O. Groene, L. Gulácsi, D. Ivankovic, T. Jansen, S. P. Johnsen, S. de Lusignan, J. Mainz, S. Nuti, N. Klazinga, on behalf of the HealthPros Network<br>Managing COVID-19 within and across health systems: why we need performance intelligence to coordinate a global response

**IDO-COVID-19** - The COVID-19 Infectious Disease Ontology (version: 8-3-2020)	Covers epidemiology, classification, pathogenesis, and treatment of terms used to represent infection by the SARS-CoV-2 virus strain, and the associated COVID-19 disease.	486	60	426	Babcock, S., Beverley, J., Cowell, L. G., & Smith, B. (2020, April 27). The Infectious Disease Ontology in the Age of COVID-19. https://doi.org/10.31219/osf.io/az6u5
**CIDO**- Coronavirus Infectious Disease Ontology (version: 1.0.114)	Developed to provide standardized human- and computer-interpretable annotation and representation of various coronavirus infectious diseases, including their etiology, transmission, pathogenesis, diagnosis, prevention, and treatment.	5156	528	4628	He, Y., Yu, H., Ong, E. *et al.* CIDO, a community-based ontology for coronavirus disease knowledge and data integration, sharing, and analysis. *Sci Data* 7, 181 (2020). https://doi.org/10.1038/s41597-020-0523-6
**CODO-** COviD-19 Ontology for Cases and Patient information	COVID-19 Ontology is a data model for publishing COVID-19 data	52	3	49	B. Dutta, M. DeBellis CODO: An Ontology for Collection and Analysis of Covid-19 Data arXiv:2009.01210v1

Supplementary Table 3: Comparison to other related ontologies (as of August 14, 2020)

Note: The comparison on concepts has been done automatically. This is not very accurate as many of the above mentioned ontologies have not imported concepts from previously existing ontologies or have not re-used the concepts. Therefore, an exact comparison of concept to concept was not feasible.

As you can see from the **Supplementary Table 3,** all ontologies are related with COVID-19 or SARS-CoV-2. However, each ontology has a very distinct scope and use cases. In the upcoming versions of our COVID-19 Ontology, we will be incorporating the missing concepts in comparison with other ontologies as well as collaborating with the other ontology development initiatives.

Text Mining use case of ontology

The ontology discussed in the paper acts as the semantic layer for the Text Mining based knowledge discovery software, SCAIview (https://www.scai.fraunhofer.de/en/business-research-areas/bioinformatics/products/scaiview.html). In the context of COVID-19, we have developed a specific instance of SCAIView (https://covid.scaiview.com/) which allows information retrieval with semantic searches in large text collections specific to COVID-19. We achieved this version of SCAIView by combining free text searches along with the concepts from COVID-19 Ontology.

Below are some of the specific applications we would like to illustrate in the SCAIView instance which uses COVID-19 Ontology.

A. Filtering the relevant documents of COVID-19 or SARS-CoV-2

The ontology discussed in the paper acts as the semantic layer for retrieving COVID-19 specific literature. It is also possible to highlight (or annotate) the COVID-19 or SARS-CoV-2 related terms in the texts. This application is available in abstracts as well as in the full text (freely available).

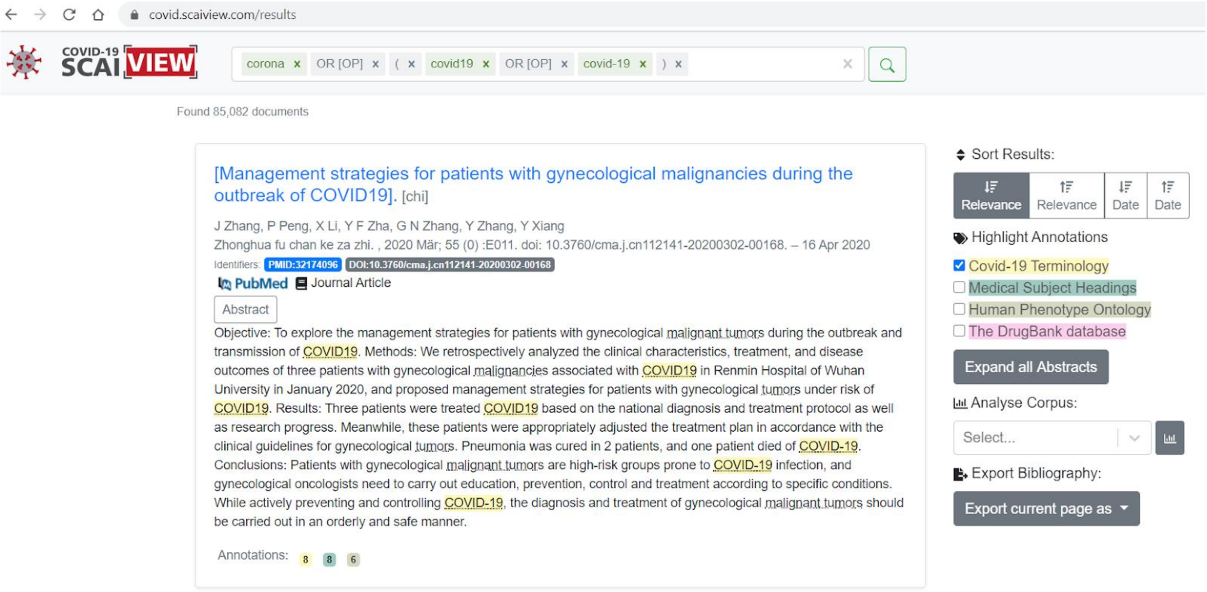

Supplementary Figure 2: SCAIView retrieves 85082 documents with the search term "*COVID-19*". The software automatically adds the synonyms of the appropriate concept to retrieve articles.

B. Data Mining / Information extraction of metadata from COVID-19 related articles

Here is an example of how COVID-19 ontology, in combination with DrugBank, could be used as a semantic layer to search and retrieve the COVID-19 or SARS-CoV-2 related drugs. SCAIView provides the lists of drugs that are mentioned in the literature in the context of COVID-19 or SARS-CoV-2, and you could download this for your further analyses.

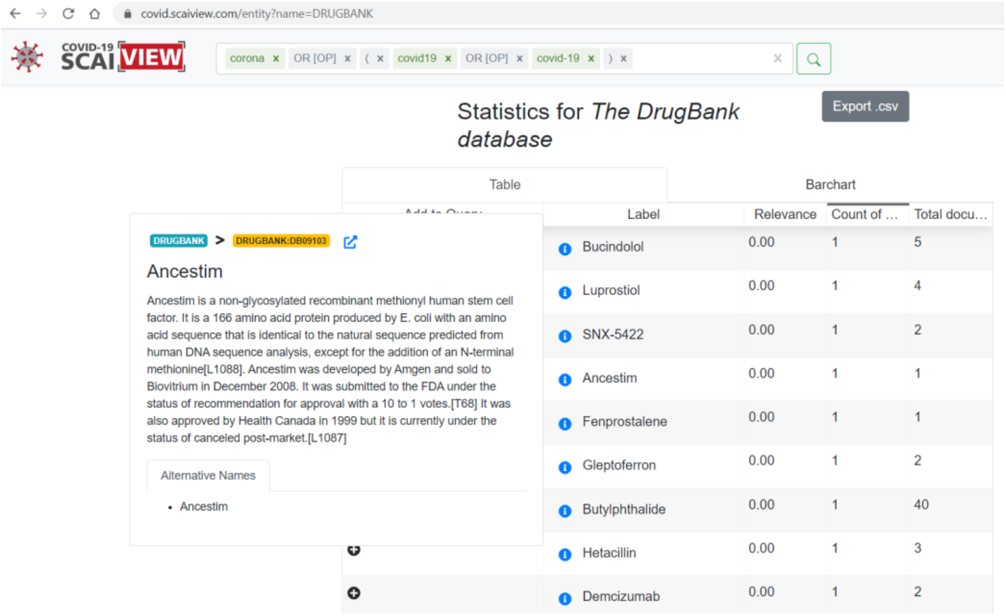

Supplementary Figure 3: SCAIView retrieves the lists of drugs based on the relevance (based on the number of times it is referred to in various COVID-19 or SARS-CoV-2 related articles).

C. Grouping COVID-19 articles into specific topics

SCAIView applies search query expansion to detect documents associated with a BIN (BIN referring to a COVID-related research topic such as 'risk factor,' 'clinical aspect' 'prevention and control', 'model', 'transmission process', 'signs and symptoms', and 'virology'). In the ontology, we have added axioms to group together the concepts into various BINs.

For a given BIN identifier, the search is augmented with all subclasses of that identifier. The HermiT (http://www.hermit-reasoner.com/) reasoner is used to include asserted as well as inferred subclasses.

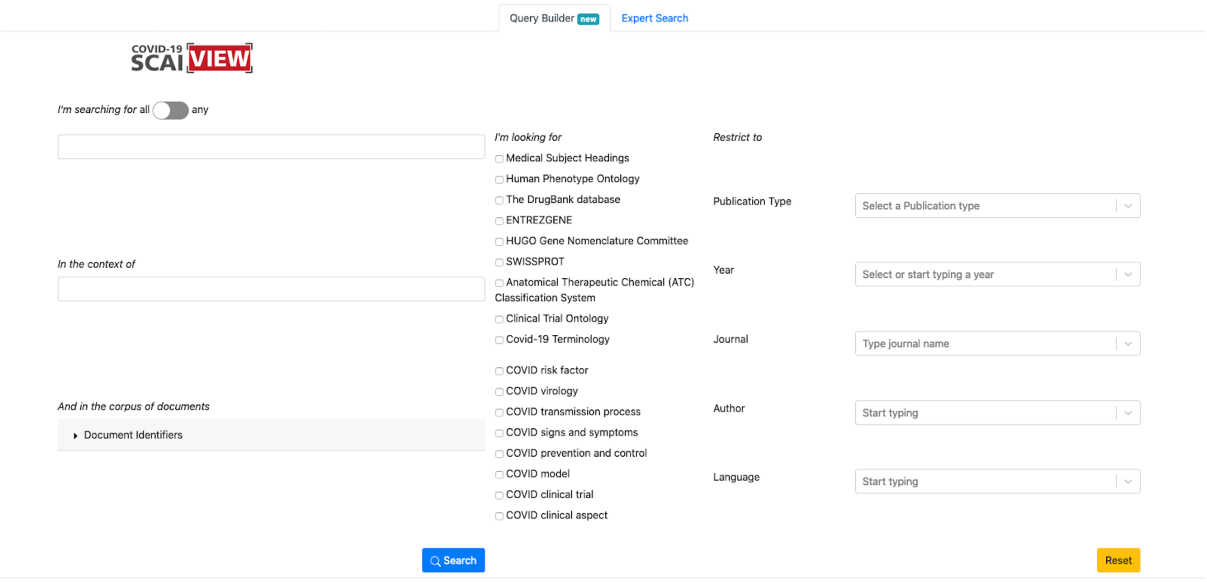

Supplementary Figure 4: The COVID-19 BINs integrated in SCAIView which retrieves articles based on COVID-19 specific topics.
